# Highly Active Thermophilic L-Asparaginase from *Melioribacter roseus* Represents a Novel Large Group of Type II Bacterial L-Asparaginases from Chlorobi-Ignavibacteriae-Bacteroidetes Clade

**DOI:** 10.3390/ijms222413632

**Published:** 2021-12-20

**Authors:** Maria Dumina, Alexander Zhgun, Marina Pokrovskaya, Svetlana Aleksandrova, Dmitry Zhdanov, Nikolay Sokolov, Michael El’darov

**Affiliations:** 1 Group of Fungal Genetic Engineering, Federal Research Center “Fundamentals of Biotechnology” of the Russian Academy of Sciences, 117312 Moscow, Russia; zzhgun@mail.ru; 2 Laboratory of Medical Biotechnology, Institute of Biomedical Chemistry, 119121 Moscow, Russia; ivan1190@yandex.ru (M.P.); v-aleksandrov@yandex.ru (S.A.); zhdanovdd@gmail.com (D.Z.); sokolov2144@yandex.ru (N.S.)

**Keywords:** L-asparaginase, thermophile, heterologous expression, biochemical properties, kinetic characteristics, cytotoxic activity

## Abstract

L-asparaginase (L-ASNase) is a biotechnologically relevant enzyme for the pharmaceutical, biosensor and food industries. Efforts to discover new promising L-ASNases for different fields of biotechnology have turned this group of enzymes into a growing family with amazing diversity. Here, we report that thermophile *Melioribacter roseus* from *Ignavibacteriae* of the Bacteroidetes/Chlorobi group possesses two L-ASNases—bacterial type II (MrAII) and plant-type (MrAIII). The current study is focused on a novel L-ASNase MrAII that was expressed in *Escherichia coli*, purified and characterized. The enzyme is optimally active at 70 °C and pH 9.3, with a high L-asparaginase activity of 1530 U/mg and L-glutaminase activity ~19% of the activity compared with L-asparagine. The kinetic parameters K_M_ and V_max_ for the enzyme were 1.4 mM and 5573 µM/min, respectively. The change in MrAII activity was not significant in the presence of 10 mM Ni^2+^, Mg^2+^ or EDTA, but increased with the addition of Cu^2+^ and Ca^2+^ by 56% and 77%, respectively, and was completely inhibited by Zn^2+^, Fe^3+^ or urea solutions 2–8 M. MrAII displays differential cytotoxic activity: cancer cell lines K562, Jurkat, LnCap, and SCOV-3 were more sensitive to MrAII treatment, compared with normal cells. MrAII represents the first described enzyme of a large group of uncharacterized counterparts from the Chlorobi-Ignavibacteriae-Bacteroidetes clade.

## 1. Introduction

L-asparaginase (EC 3.5.1.1; L-asparagine amidohydrolase) (L-ASNase) is a biotechnologically relevant enzyme used in the pharmaceutical, biosensor and food industries [1,2,3]. It catalyzes the conversion of the amino acid L-asparagine (L-Asn) to L-aspartic acid and ammonia [4]. The enzyme is firstly known for its anticarcinogenic properties. L-ASNase selectively targets the metabolism of susceptible cancer cells by exploiting deficiencies in metabolic pathways. The enzyme exhibits a depletion effect on the concentration of L-Asn in the extracellular fluid. A diminished level of L-Asn and the inability of susceptible tumor cells to synthesize their own L-Asn leads to the inhibition of protein synthesis, cell cycle arrest in the G1 phase, and ultimately apoptosis in leukemic cells [5,6].

The enzyme is used in the food industry as a processing agent to reduce the acrylamide levels in commercial fried foods [2,7]. L-ASNase catalyzes the hydrolysis of L-Asn, not allowing the reaction of reducing sugars with this amino acid for the generation of acrylamide, classified as “reasonably anticipated to be a human carcinogen” [8].

The application of L-ASNase in biosensors allows monitoring L-Asn levels both for diagnostic purposes and the food industry [9,10,11,12].

Continuous research efforts to discover new promising L-ASNases for different fields of biotechnology have turned this group of enzymes into a growing family with amazing diversity. L-ASNases have been isolated from many archaea, bacteria, fungi, and yeast [3,13], and L-ASNases of plant and mammalian origin are now also known [14,15,16].

According to the classification historically applied, L-ASNases are divided into three major groups: bacterial-type L-asparaginases, plant-type L-asparaginases, and Rhizobial-type L-asparaginases [13].

Bacterial L-ASNases are subdivided into type I and type II [17]. Type I L-ASNases are involved in nitrogen metabolism and appear to be expressed constitutively [18]. These cytoplasmic enzymes show low affinity towards L-Asn, with K_M_ values in the millimolar range [17]. On the other hand, bacterial type II L-ASNases appear to participate in carbon metabolism and their expression is tightly regulated by different factors [18]. Type-II enzymes are located in the periplasmic space. They exhibit high specific activity against L-Asn with K_M_ values in two orders of magnitude lower (micromolar K_M_) compared to bacterial type I L-ASNases [13].

Plant-type L-ASNases or type III L-ASNases appear to play the dominant role in asparagine degradation, by hydrolyzing the side-chain amide bond of L-Asn or its β-peptides in a significant fraction of higher plants [19]. These enzymes belong to the superfamily of N-terminal nucleophile hydrolases [17]. Two forms of plant-type L-asparaginases are described, potassium-dependent and potassium-independent, which are immunologically distinct. High K_M_ values (millimolar) of these enzymes restrict their application in biomedicine [17,19,20].

Rhizobial-type L-ASNases include L-ASNases displaying sequence homology with L-ASNase from the soil-living bacteria *Rhizobium etli* [19]. In *R. etli*, two asparaginase activities were identified: a thermostable and constitutive asparaginase activity (asparaginase I); and asparaginase II, characterized by its thermolability, induced by asparagine, and repressed by the carbon source [19]. The properties of *R. etli* L-ASNases have yet to be elucidated. However, preliminary kinetic data indicate that these enzymes display submillimolar K_M_ and no glutaminase activity, and thus can be considered as potential antileukemic agents [21].

Despite widespread acceptance, this classification, which is based on the source of the first enzymes that were discovered, may be misleading. L-ASNases of the three canonical types, i.e., bacterial-type, plant-type and *R. etli*-type, are found throughout several kingdoms of life. For example, *Escherichia coli* possesses not only bacterial type I (EcAI, encoded by the *ansA* gene) and type II (EcAII, *ansB*) L-ASNases but also plant-type L-ASNase—EcAIII (*ybiK*) [17,22]. Plant-type L-ASNases are also found in insects and mammals [23,24].

To correct the situation, Schultz da Silva et al. have recently proposed to remove the class name, and rename bacterial-type L-asparaginases, plant-type L-asparaginases, and Rhizobial-type L-asparaginases as Class 1, Class 2 and Class 3, respectively [25].

Here, we identified that *Melioribacter roseus*, belonging to a novel proposed phylum *Ignavibacteriae* of the Bacteroidetes-Chlorobi group [26], possesses two L-ASNases—Class 1 (known as bacterial-type) and Class 2 (plant-type)—that share a high similarity with the respective counterparts originating from other members of the Bacteroidetes-Chlorobi group. Both Class 1 and Class 2 enzymes were identified in the genomes of most of these microorganisms. According to previous proteomic data, these bacteria are engaged in biofilm-derived organic matter degradation, through high expression of hydrolases and, in particular, L-asparaginases [27]. However, none of these L-ASNases have been characterized to date.

*M. roseus* itself is a recently described moderately thermophilic, facultative anaerobic chemoorganotrophic bacterium, isolated from a microbial mat under the flow of hot water (46 °C) from a 2775-m-deep oil exploration well (Russia) [26].

Among the two annotated in the genome of *M. roseus* L-ASNases, Class 2 (plant-type) L-ASNase (MrAIII) and Class 1 (bacterial type II) L-ASNase (MrAII), MrAII was chosen for further detailed elucidation, as currently only bacterial type II enzymes are approved for biomedical application.

The present study focuses on MrAII biochemical characterization and preliminary assessment of in vitro cytotoxic activity to evaluate its potential as an anticancer agent. MrAII represents the first described enzyme of a large group of closely related bacterial type II L-ASNases, originating from the members of the Bacteroidetes-Chlorobi group.

## 2. Results

### 2.1. Identification and Sequence Comparison of L-Asparaginases from M. roseus

The genomic sequence of the moderately thermophilic bacterium *M. roseus* P3M-2 has been completed and deposited as GenBank accession number NC_018178.1 [26,28]. Bioinformatics analysis of the available whole genome sequence revealed that *M. roseus* P3M-2 possesses two enzymes exhibiting L-asparaginase activity—WP_014855981.1 and WP_014855710.1—encoded by the MROS_RS06765 and MROS_RS05340 genes, respectively.

Homology search demonstrated that putative isoaspartyl peptidase/L-asparaginase WP_014855981.1 shared the highest identity with the previously characterized isoaspartyl peptidase/L-asparaginase from *E. coli*—WP_146849425.1—with 55.2% classified as plant-type L-ASNase EcAIII. When the amino acid sequence of WP_014855981.1 was aligned with the known plant-type L-ASNases, all previously identified catalytic residues, including the nucleophilic threonine residue, involved in autocleavage and catalysis, followed by a conserved pentapeptide (VGAVA), were found to be conserved in all the sequences [29] (Supplementary Material, Figure S1). Thus, enzyme WP_014855981.1 of *M. roseus* represents plant-type (Class 2) L-asparaginase—MrAIII. However, MrAIII shares ~40% identity with other characterized plant-type L-ASNases from *Thermococcus kodakarensis* (Q5JHT1.1) and *Pyrobaculum calidifontis* (ABO08395.1), with 43.1% and 39.5%, respectively.

**Figure 1:**
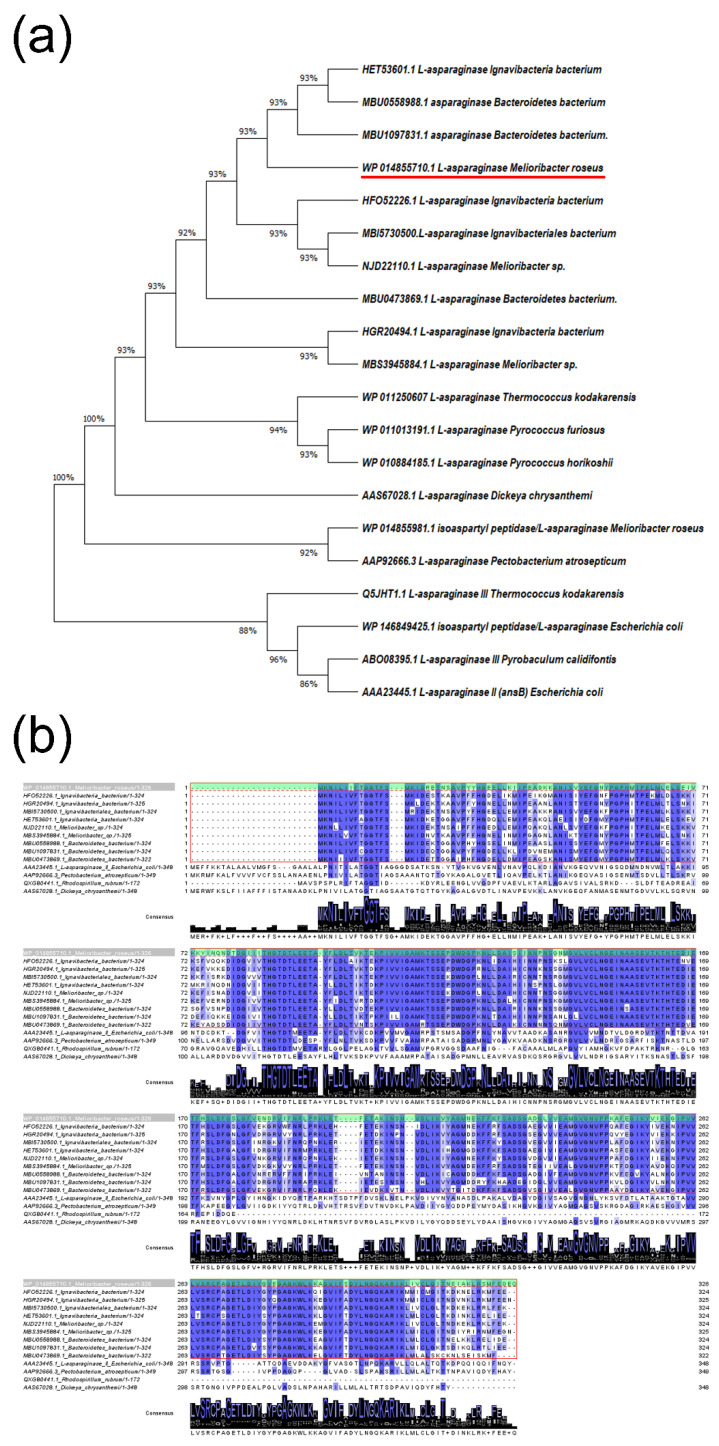
Comparison of L-asparaginases (L-ASNases) from *M. roseus*. (**a**) Molecular phylogenetic analysis of selected L-ASNases using the minimum evolution method [37]. The analysis involved 20 amino acid sequences. There were a total of 349 positions in the final dataset. Evolutionary analyses were conducted in MEGA X [38]. (**b**) Multiple sequence alignment of type II L-ASNase from *M. roseus* (MrAII) (WP_014855710.1, marked with red dashed line and green box) with its homologs from the members of the Bacteroidetes-Chlorobi group, marked with a red box and characterized bacterial type II L-ASNases from *E. coli* (AAA23445.1), *P. atrosepticum* (AAP92666.3), *R. rubrum* (QXG80441.1), and *D. chrysanthemi* (AAS67028.1). Identical amino acid residues are marked by blue. Multiple sequence alignments were carried out using Clustal version 2.1 [39].

The second identified L-ASNase in the *M. roseus* P3M-2 genome WP_014855710.1 was classified as bacterial L-ASNase type II (Class 1) (MrAII). The amino acid sequence comparison showed that MrAII displayed high homology with the wide group of uncharacterized L-ASNases from recently annotated genomes of *Ignavibacteria* bacterium (HGR20494.1, HFO52226.1, MBI5730500.1, HET53601.1) [30,31]—80.3–79.0%, *Melioribacter* sp. (NJD22110.1, MBS3945884.1)—80.5–78.3%, and *Bacteroidetes* bacterium (MBU0558988.1, MBU1097831.1, MBU0473869.1)—78.7–76.1% identity. However, the level of homology between MrAII and known type II L-ASNases, including those of FDA-approved for use as antileukemic agents, is rather low—lower than between MrAII and MrAIII (44.4%). The identity of the putative type II L-ASNase from *M. roseus* and well-characterized periplasmic L-ASNases from *Pectobacterium atrosepticum* (*Erwinia carotovora* subsp. atroseptica) (GenBank accession No. AAP92666.3) [32,33] and *Rhodospirillum rubrum* (GenBank accession No. QXG80441.1) [34,35,36] is about 32.7% and 41.7%, respectively. The identity between MrAII and FDA-approved L-ASNases from *E. coli* (UniProtKB accession number P00805, marketed under the brand name Elspar, GenBank accession No. AAA23445.1) and *Erwinia chrysanthemi* (*Dickeya chrysanthemi*, *Pectobacterium chrysanthemi*) (UniProtKB accession number P06608, marketed under the brand name Erwinaze, GenBank accession No. AAS67028.1) is only 35.2% and 32.4%, respectively. Interestingly, the identity between thermophilic MrAII and bacterial type I L-ASNases with known structures from other thermophiles *Thermococcus kodakarensis* KOD1 (WP_011250607], *Pyrococcus furiosus* (WP_011013191.1), *Pyrococcus horikoshii* (WP_010884185.1) is 26.7–26.5%. The data indicate the low overall sequence similarity of MrAII with other previously characterized L-ASNases, of various origins and types (Figure 1).

Among two annotated in the genome of *M. roseus* L-ASNases—plant-type L-ASNase MrAIII (Class 2) (WP_014855981.1) and bacterial L-ASNase type II MrAII (Class 1) (WP_014855710.1)—MrAII was chosen for further detailed elucidation, as currently only bacterial type II enzymes are approved for biomedical application.

### 2.2. Gene Cloning, Expression and Recombinant Enzyme Purification

The native type II L-asparaginase gene from *M. roseus* (*mrAII*), which consists of 981 nucleotides corresponding to a polypeptide of 327 amino acids, was artificially synthesized and cloned into the pET-28a(+) vector under the control of the T7 promoter.

The constructed plasmid was transformed into the host *E. coli* BL21 (DE3) for the heterogeneous expression of MrAII. Enzyme purification of MrAII was achieved by ion-exchange chromatography with a final yield of 75.5% (Table 1).

**Figure 2:**
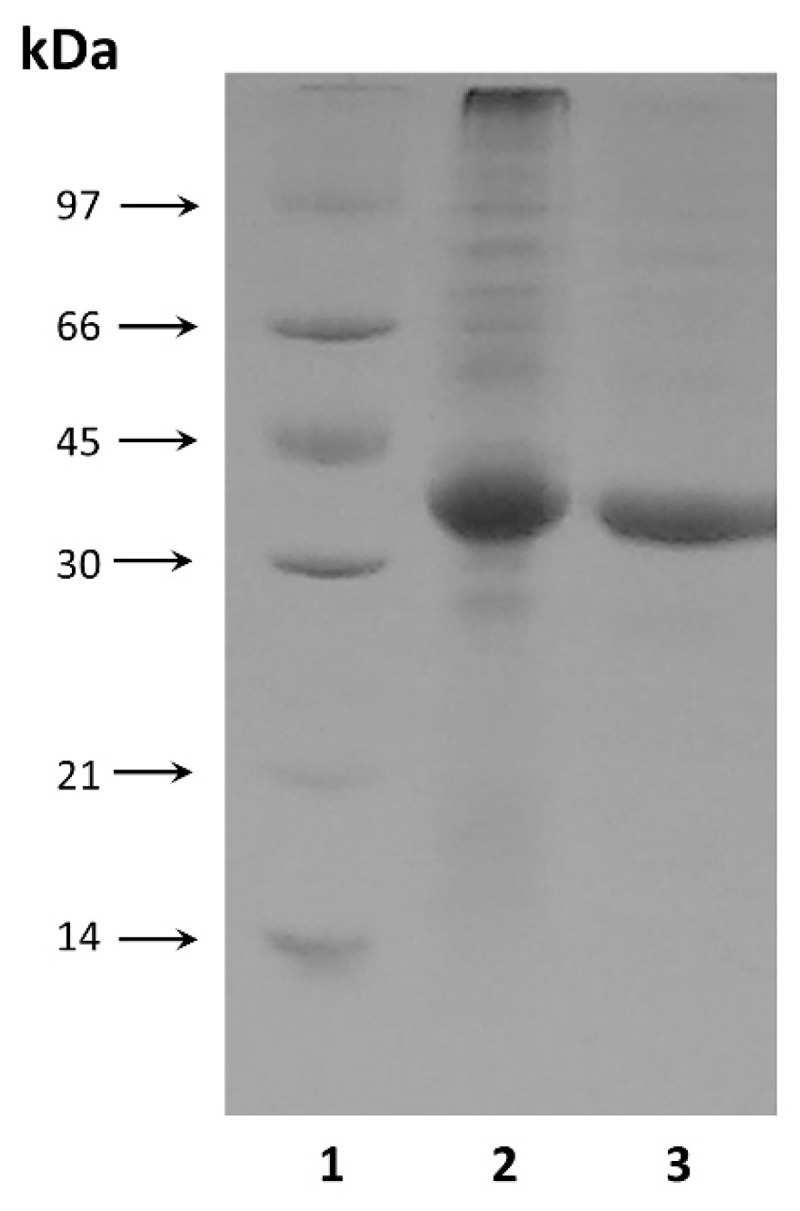
SDS-PAGE analysis: 1—protein molecular weight marker; 2—MrAII in cell free homogenate at a concentration of 20 µg; 3—purified MrAII at a concentration of 11 µg.

The purified enzyme showed electrophoretic homogeneity with a single major band on the SDS-PAGE (Figure 2). The molecular weight of the purified enzyme was estimated to be approximately 34.5 kDa by SDS-PAGE (Figure 2), which was consistent with the theoretical value calculated from the amino acid sequence of the processed enzyme form (35.3 kDa).

### 2.3. Specific Activity of MrAII and Enzyme Kinetics

The recombinant type II L-ASNase from *M. roseus* exhibited high hydrolysis activity toward L-Asn with a specific activity of 1530 U/mg. Substrate specificity experiments demonstrated that MrAII exhibits additional L-glutaminase activity but no D-asparaginase activity. The enzyme could utilize L-glutamine as a substrate, with approximately 19% of the enzyme activity when L-Asn was used as the substrate.

The kinetic properties of the recombinant type II L-ASNase from *M. roseus* were assessed. V_max_ was found to be 5573 µM/min. The K_M_ for the substrate L-Asn was estimated to be 1.4 mM for MrAII.

### 2.4. Effect of Temperature, pH and Metal Ions on Enzyme Activity. Chemical Stability of MrAII

**Figure 3:**
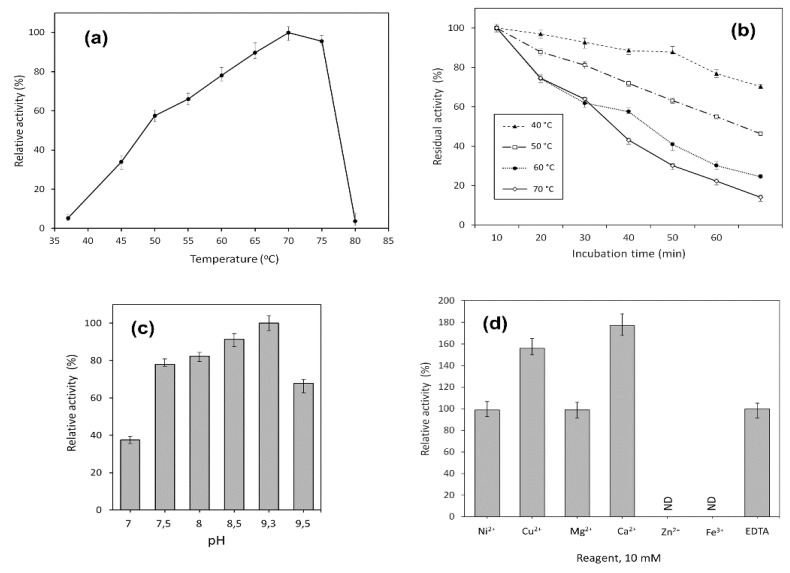
Effect of temperature on (**a**) activity, and (**b**) stability of MrAII. Stability is expressed as residual activity (%) after 0–60 min of incubation. Effects of (**c**) pH, and (**d**) various metal cations, on the catalytic activity of the MrAII; ND, not defined.

The activity of MrAII was evaluated at different temperatures, ranging from 37 to 80 °C. It was shown that the enzyme exhibited high activity in a range of temperatures from 60 to 75 °C, with a maximum at 70 °C (Figure 3a). The time course of thermal inactivation of purified L-asparaginase is shown in Figure 3b.

Significantly, MrAII retained more than 70% of its initial activity after 60 min incubation at 40 °C, close to the physiological temperature (Figure 3b).

The enzymatic activity of the purified enzyme was monitored in different pH systems, ranging from pH 4.0 to 9.5. MrAII exhibited maximum activity at pH 9.3, and only 20% of its at acidic pH was less than 7.0. The pH-dependent activity profile of MrAII in a working range of pH 7.0–9.5 is given in Figure 3c. According to experimental data, the recombinant enzyme retained high relative activity in a range of pH 7.5–9.5, independent of the buffer system.

The effect of various metal cations Ni^2+^, Cu^2+^, Mg^2+^, Zn^2+^, Ca^2+^, Fe^3+^ and EDTA added at a concentration of 10 mM on MrAII activity was examined (Figure 3d). No significant change in the enzyme activity was observed in the presence of Ni^2+^, Mg^2+^, or EDTA. The addition of Cu^2+^ and Ca^2+^ resulted in a 56% and 77% increase in MrAII activity, respectively. On the other hand, Zn^2+^ and Fe^3+^ completely inhibited the activity of the enzyme (Figure 3d).

The same results were obtained in the presence of 2–8 M urea solutions: MrAII completely lost its activity at all concentrations tested.

### 2.5. Determination of MrAII Cytotoxic Activity

**Figure 4:**
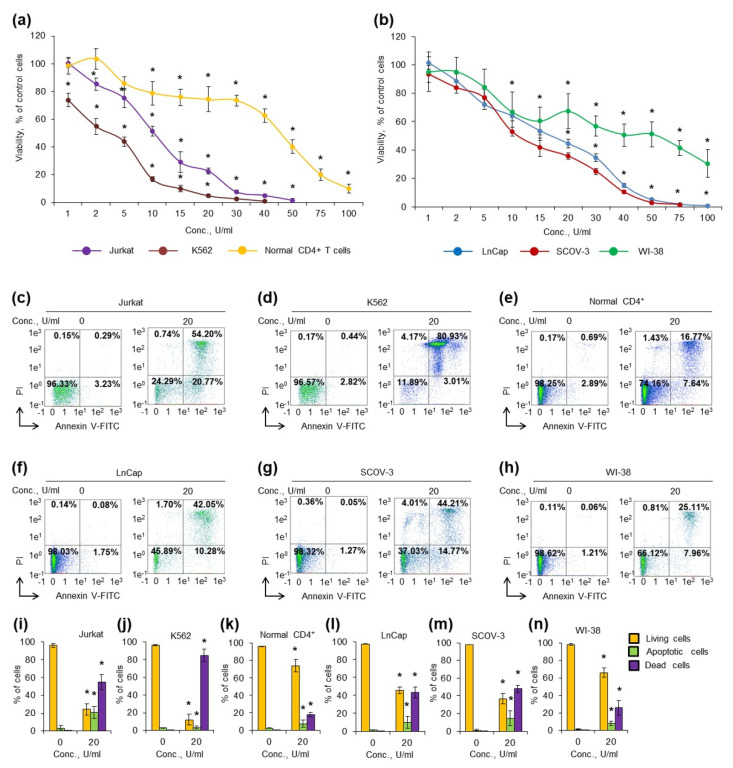
Cytotoxic activity MrAII against cancer cells. Cancer and normal cells were cultivated for 72 h in the presence of different concentrations of the enzyme. (**a**) Cell viability measured by the MTT test for cancer and normal T lymphocytes; (**b**) cell viability measured by MTT test for solid tumor cells and fibroblasts; (**c**–**h**) representative flow cytometry diagrams for cells incubated with 20 U/mL of the enzyme and labeled with annexin V-FITC and PI. Ratios of living cells (lower left quadrants), apoptotic cells (lower right quadrants) and dead cells (two upper quadrants) are presented. (**i**–**n**) Histograms of live, apoptotic and dead cells incubated with 20 U/mL of the enzyme. Conc., concentration. PI, propidium iodide. *n* = 4. * *p* ≤ 0.05 vs. control untreated cells.

To test the cytotoxic activity of MrAII, different types of human leukemic and solid tumor cancer cells along with normal fibroblasts and normal CD4+ T cells were cultivated in the presence of different concentrations of the enzyme. Cell viability and apoptosis induction were measured after 72 h of incubation (Figure 4, Supplementary Material, Figure S2). Table 2 represents the IC_50_ and IC_90_ values for the tested cell lines.

The K562 cell line was the most sensitive among cancer cells, and the enzyme was able to significantly decrease cell viability even at the lowest concentration 1 U/mL (Figure 4a). Normal CD4+ T cells were more resistant than cancer lymphocytes, and a significant decrease in cell viability was observed at concentrations higher than 10 U/mL. Both solid tumor cell lines LnCap and SCOV-3 demonstrated almost equal sensitivity to the enzyme (Figure 4b). Normal human fibroblast WI-38 cells were also sensitive to low concentrations of the enzyme (1–15 U/mL); however, they appeared to be more resistant to higher concentrations (30–100 U/mL).

The results demonstrated that MrAII has cytotoxic activity and can induce apoptosis in cancer cells, whereas high concentrations were necessary for the induction of apoptosis in normal cells.

## 3. Discussion

The increasing need for more robust industrial L-ASNases attracts much attention to discovering new potential sources of the enzyme.

Recent proteomic data indicate that the members of the Bacteroidetes-Chlorobi group are engaged in biofilm-derived organic matter degradation, in particular, through high expression of asparaginases [27]. Nevertheless, the enzymes originating from microorganisms of this group still remain uncharacterized. In this report, we focused on the characteristics of L-ASNase originating from *M. roseus* belonging to the class *Ignavibacteria* Bacteroidetes-Chlorobi group.

Bioinformatics analysis revealed that *M. roseus* possesses two enzymes exhibiting L-asparaginase activity that were identified as plant-type (Class 2) (MrAIII) and bacterial type II (Class 1) (MrAII) L-ASNases, based on the amino acid sequence analysis.

The role of plant-type L-ASNases observed in microorganisms, insects, and mammals, is not obvious, especially in view of the presence of other types of the enzyme in these organisms. Plant-type enzymes have dual isoaspartyl aminopeptidase/L-asparaginase activity [17].

The first (L-asparaginase) activity is essential in plant metabolism [17]. Asparagine has an N:C ratio of 2:4 (in contrast to 2:5 for glutamine), which makes it an efficient molecule for the storage and transport of nitrogen in living organisms. The atmospheric nitrogen assimilated in the roots of plants is transported to metabolically active parts (shoots, leaves, and flowers) in the form of L-Asn, where it is liberated by hydrolysis. Thus, the metabolism of plants requires efficient l-asparaginase activity in fast growing tissues [17]. The second (isoaspartyl aminopeptidase) activity of plant-type L-ASNases protects dormant seeds from the spontaneous accumulation of highly toxic β-asparagine dipeptides [17].

In *M. roseus*, MrAIII appears to be involved in nitrogen metabolism in the absence of type I L-ASNase and in the degradation of β-aspartyl-containing proteins.

On the other hand, type II L-ASNases appear to participate in carbon metabolism, and their expression is tightly regulated by different factors [18,40]. Apparently, MrAII plays an essential role in the growth of the facultative anaerobe *M. roseus* under oxygene-limiting conditions. In the study using type II L-ASNase from *E. coli* (EcAII), it was confirmed that EcAII was activated by anaerobiosis (by fumarate and nitrate reductase and cyclic AMP receptor) [41]. Recent data suggest that EcAII enables anaerobic respiration and is part of a metabolic pathway leading to the production of fumarate as a terminal electron acceptor. EcAII is localized in the periplasm to be able to utilize exogenous (extracellular) L-Asn to produce fumarate, as under anaerobic conditions transport of L-Asn from the cytoplasm to the periplasm is abolished [21,40].

As previously reported, the L-ASNases enzyme “set” may be different for species of the genus [18]. Some can possess L-asparaginases of two (several) types, whereas others possess only one type. Nevertheless, both type II and type III L-ASNases are annotated in other recently deposited in GenBank genomes, not only *Melioribacter* sp. (GenBank accession No. VEPI00000000.1, JAGXSM000000000.1), but also *Ignavibacteria* bacterium (GenBank accession No. DSRE00000000.1, DSBF00000000.1), and *Bacteroidetes* bacterium (GenBank accession No. JAHJCD000000000.1, JAHITB000000000.1, JAHJDY000000000.1). Sequence identity for bacterial type II L-ASNases of these microorganisms is not less than 76% (80.5–76.1%), and type III L-ASNases not less than 54% (54.0–71.7%). Thus, enzymes from *M. roseus* MrAII and MrAIII represent a large group of uncharacterized type II and type III L-ASNases, respectively, originating from the members of the Bacteroidetes-Chlorobi group. These enzymes have no significant sequence similarity with the thoroughly studied counterparts.

Despite the importance of both L-ASNases MrAII and MrAIII, MrAII was chosen for further detailed elucidation as having higher potential for future biomedical applications.

*M. roseus* is a moderately thermophilic bacterium isolated from the flow of hot water (46 °C) [26]. To adapt to elevated habitat temperatures, thermophilic L-asparaginases acquired a number of specific features, in particular, a low occurrence of thermolabile residues [42]. A correlation between thermostability and the ratio of preferred (Glu and Lys) and avoided (Gln and His) amino acids was reported [43,44].

The amino acid sequence analysis revealed that the Glu and Lys content was 16.6%, whereas the Gln and His content was 2.5% in MrAII. The enzyme contained 38.5% of the preferred thermostable protein Ala, Gly, Glu, Val, and Lys residues.

According to experimental data, MrAII exhibits maximum activity at 70 °C and pH 9.3. Similar results were reported for L-ASNases from the thermophilic bacteria *Streptomyces thermoluteus* subsp. fuscus NBRC 14270, *Thermus thermophilus*, and *Thermus aquaticus* T351 with the optimum of temperature 63.6–75.0 °C, and pH 8.0–9.5 [3].

As a rule, thermophilic L-ASNases exhibit higher L-asparaginase activity compared with mesophilic counterparts [3,45]. The same was observed for MrAII. The recombinant type II L-ASNase from *M. roseus* hydrolyzed L-Asn with a specific activity of 1530 U/mg. According to experimental data, MrAII is more active not only than mesophilic L-ASNases from *E. coli*, *Erwinia carotovora*, *Bacillus subtilis* and *Wolinella succinogenes*, with specific activities of 235, 430, 92, and 200 U/mg, respectively [45], but it has the highest activity reported so far for thermophilic L-ASNases of bacterial origin. In previous studies, L-ASNase activity for the thermophilic bacteria *S. thermoluteus* subsp. fuscus NBRC 14270, *T. thermophilus* and *T. aquaticus* T351, was estimated to be 68.09, 840, and 585 U/mg, respectively [3].

The kinetic properties of the recombinant type II L-ASNase from *M. roseus* were found to be V_max_ 5573 µM/min and K_M_ 1.4 mM. The K_M_ value for the enzyme was quite high compared to those of the non-thermophiles: *E. coli*—0.018 mM; *E. carotovora*—0.085–0098 mM; *B. subtilis*—0.43 mM; *W. succinogenes*—0.048 mM [45]. On the other hand, it was lower compared with the L-ASNases from the thermophilic bacteria: *S. thermoluteus* subsp. fuscus NBRC 14270—1.83 mM; *T. thermophilus*—2.8 mM; *T. aquaticus*—8.6 mM [3].

Increased K_M_ values have previously been demonstrated for bacterial and eukaryotic enzymes originating from thermophilic microorganisms. The K_M_ values were higher for thermophilic homologs than mesophilic ones, for enzymes such as phosphoglycerate kinase [46], glutamate dehydrogenase [47], alkaline phosphatase [48,49,50], GTPase (TrmE) [51] and glucose-6-phosphate dehydrogenase [52].

It appears that adaptation at high environmental temperatures involves an increase in K_M_ and k_kat_ for thermophilic L-ASNases [53]. This characteristic feature allows optimizing catalytic efficiency by reaching a balance between substrate binding and the rate of product release.

In contrast to some L-ASNases that retain significant structural stability under denaturing conditions [3], MrAII completely lost its activity in the presence of urea solutions at 2–8 M. 

The non-thermophilic bacterial L-ASNase II thermostability is poor compared with the thermophilic bacterial L-ASNase. When incubated at 70 °C for 30 min, 80% of *E. coli* L-asparaginase II activity was lost [54]. For MrAII, this figure was 42.4%. The enzyme retained more than 70% of its initial activity after 60 min incubation at 40 °C, close to physiological temperature.

The addition of Cu^2+^ or Ca^2+^ resulted in a 56% or 77% increase in MrAII activity, respectively. Previously, it was shown that some metal cations, including Ca^2+^, binding to a protein molecule, played an important role in stabilizing the structure, thus preventing denaturation at high temperatures [3].

The main field of biotechnology application for L-asparaginases is anticancer therapy [55,56]. Here, we have performed an attempt to evaluate the anticancer potential of MrAII.

To explore the cytotoxic activity in vitro, we incubated cells with 20 U/mL of the enzyme and measured the induction of apoptosis by the labeling of phosphatidylserine on cell membranes with annexin V-FITC and cell DNA by PI, followed by flow cytometry. The results of apoptosis measurement were in a good agreement with the results from the MTT-test. The enzyme induced apoptosis more efficiently in Jurkat, or K562 cancer lymphocytes: less than 12% or 25% of them remained alive after incubation, respectively (Figure 4c,d,i,j). Solid tumor cells LnCap and SCOV-3 cells appeared to be the most resistible, with 46 and 38% of living cells remaining alive, respectively (Figure 4f,g,l,m). The enzyme induced apoptosis in normal CD4+ T cells, and normal fibroblast at a concentration of 20 U/mL; however, cell viability was much higher than in tumor cells (Figure 4e,h,k,n). The results demonstrated that the enzyme has cytotoxic activity, and can induce apoptosis in cancer cells, whereas high concentrations are necessary for the induction of apoptosis in normal cells. The difference in the sensitivity to the enzyme between normal and cancer cells can become a basis for the further development of this enzyme as an antitumor drug.

Two ways of future MrAII biomedical application can be considered: as a single anticancer agent, and as a part of therapy in combination with glutaminase-free asparaginase, due to exhibited additional glutaminase activity.

Until recently, it was believed that the L-glutaminase activity exhibited by L-ASNases was highly undesirable, as it contributes to the toxic side effects of the enzyme treatment [57,58]. Thus, the research community was actively pursuing the search for glutaminase-free, or the development of glutaminase-deficient L-ASNase variants [5,57,59,60].

However, advancements in cancer cell metabolism suggest that the picture is much more complex. Recent data demonstrate that L-asparaginase activity alone may not be sufficient for L-ASNase cytotoxicity, and that glutaminase activity may be required for full antitumor efficacy against cancer [58,61,62,63].

Cancer cells are capable of reprogramming the survival amino acid metabolism by using compensatory pathways via transcriptional, epigenetic, and post-translational mechanisms [64]. Both L-glutamine and L-asparagine are essential for the growth of highly proliferative cells, and the anticancer activity of L-ASNase can require the enzyme’s glutaminase activity [61,62,64].

A key player in driving the resistance to L-ASNase treatment is the expression of asparagine synthetase (ASNS), the rate-limiting enzyme for de novo biosynthesis of L-asparagine [65,66,67].

In addition to the expression of ASNS itself, studies suggest that the availability of aspartate and glutamine, two indispensable substrates of ASNS, are also important for the cellular adaptation to the depletion of exogenous asparagine [67,68]. In cell culture conditions, most cancer cells can use glutamine to synthesize aspartate de novo.

The role of the tumor microenvironment in resistance to anticancer therapy has also been highlighted in recent years [69,70]. Related to L-ASNase treatment tolerance, it was shown that bone marrow adipocytes can protect cells of acute lymphoblastic leukemia from L-ASNase-induced cytotoxicity by exploiting the mechanism dependent on glutamine secretion [71].

Moreover, further research is required to investigate the role of aspartate and/or glutamine availability in L-ASNase resistance; in a series of recent works it was revealed that glutaminase activity of L-ASNase can contribute to the overall efficiency of the enzyme treatment, both in vitro and in vivo [58,61,62,63].

Parmentier et al. have reported that 9 of 11 tested human leukemia cell lines were more sensitive to L-ASNases with higher glutaminase activities [58]. The same results were obtained for two patient-derived ALL samples.

According to Chan et al., L-ASNase glutaminase activity contributed to durable anticancer activity, whereas L-ASNase activity alone yielded a mere growth delay [61]. In vivo studies demonstrated that glutaminase-deficient L-ASNase was insufficiently active to prevent the recurrence of leukemia. In contrast, ASNase with glutaminase activity at the same dose, in terms of asparaginase activity, yielded a durable response. The greater extent of L-Asn depletion was revealed for L-ASNase that exhibited glutaminase activity. The effect was presumably due to the additional depletion of glutamine, which is a substrate for the synthesis of L-Asn by ASNS in cells throughout the body [61].

Additionally, it is supposed that L-ASNase glutaminase activity can be associated with the suppression of ASNS upregulation [62].

In summary, considering the risk of toxic effects associated with L-ASNase glutaminase activity, a balance is needed between the asparaginase/glutaminase activity of the enzyme to provide prolonged anticancer activity with minimal adverse reactions. MrAII exhibits additional glutaminase activity that can be important to overpower the supportive microenvironment of cancer cells.

Thus, the novel bacterial type II L-ASNase from *M. roseus* has both fundamental and applied value. MrAII expands the spectrum of available L-ASNases and represents the first described enzyme from a large group of closely related counterparts from the members of the Bacteroidetes-Chlorobi group. MrAII seems to be promising for further investigation and biotechnology application in view of the highest L-ASNase activity reported so far for enzymes of bacterial origin, in combination with increased thermal stability and cytotoxic activity toward cancer cells.

## 4. Materials and Methods

### 4.1. Enzymes and Chemicals

All chemicals used in the experiments were of analytical grade and purchased from Fluka Chemical Corp. (Fluka Chemie GmbH, Buchs, Switzerland), Merck (Merck Millipore, Darmstadt, Germany), Bio-Rad (1000 Alfred Nobel Drive, Hercules, CA, USA), Reanal (Reanal Finechemical Private Ltd., Budapest, Hungary), Serva (SERVA Electrophoresis GmbH, Heidelberg, Germany), Paneco (Moscow, Russia) or Reachem (Moscow, Russia). Enzymes were purchased from SibEnzyme (SibEnzyme-M, Moscow, Russia).

Growth media for human cancer cell lines were produced by Gibco (Thermo Fisher Scientific Inc., Waltham, MA, USA). Medium RPMI 1640 was supplemented with fetal bovine serum (Thermo Fisher Scientific Inc., Waltham, MA, USA), anti-CD28 (eBioscience Inc., San Diego, CA, USA), anti-CD3 mAbs (MedBioSpectr, Moscow, Russia), and recombinant human interleukin-2 (rHu IL-2) (R&D Systems, Minneapolis, MN, USA).

Additionally, 3-(4,5-Dimethyl-thiazol-2-yl)-2,5-diphenyltetrazolium bromide, trypsin, and PBS were used in the cytotoxicity assay, and were purchased from Serva (SERVA Electrophoresis GmbH, Heidelberg, Germany), Thermo Fisher Scientific Inc. (Waltham, MA, USA), and Paneco (Moscow, Russia), respectively.

### 4.2. Strains and Cell Lines

*E. coli* XL1-Blue (Stratagen, La Jolia, CA, USA) and *E. coli* BL21 (DE3) (Novagen, Madison, WI, USA) were used for plasmid amplification and expression, respectively.

Human chronic myelogenous leukemia K562, acute T cell leukemia Jurkat, prostate carcinoma LnCap, ovary adenocarcinoma SCOV-3 cell lines and normal human fibroblasts WI-38 (all from ATCC, Manassas, VA, USA) were used for the evaluation of L-ASNase cytotoxic activity.

### 4.3. Cloning of MrAII Coding Sequences

A putative gene predicted to encode L-ASNase MROS_RS05340 (*mrAII*) (sequence 1199322-1200302 https://www.ncbi.nlm.nih.gov/nuccore/397689003 (accessed on 15 October 2021), protein GenBank accession No. WP_014855710.1) flanked by the restriction sites *Nhe*I/*Sal*I was artificially synthesized by TWIST Bioscience (Twist Bioscience HQ, San Francisco, CA, USA). The synthesized gene was hydrolyzed and cloned into the *Nhe*I/*Sal*I digested vector pET-28a(+) under the control of the T7 promoter. The constructed vector was transformed and expressed in *E. coli* BL21 (DE3).

### 4.4. Expression and Purification of Recombinant MrAII

The selected recombinant *E. coli* clones were grown as previously described [34,72,73]. Kanamycin 0.05 mg/mL was added into the medium for the cultivation of cells harboring plasmids. Target protein expression was induced by lactose added to the expressed culture at a density of A600 1.9 to a final concentration of 0.2%. The cells were grown for an additional 17–20 h, and pelleted by centrifugation at 4000× *g* for 15 min.

All enzyme purification stages were performed at +4 °C. Five grams of biomass were suspended in 50 mL of buffer (20 mM sodium phosphate buffer pH 7.2, 1 mM glycine, 1 mM EDTA) and destroyed by ultrasound treatment [34]. Cell debris and unbroken cells were removed by centrifugation (35,000× *g*, 30 min). The supernatant, containing the enzyme, was applied to a Q-Sepharose column. Protein was eluted with a linear gradient of 0–1.0 M NaCl. Column fractions, containing enzyme (0.46–0.7 M NaCl), were collected. Ultrafiltration, desalting and buffer exchange were performed using Amicon membranes (Millipore, Burlington, MA, USA), as described previously [74]. Samples were frozen and stored at −20 °C.

Protein concentration was determined by the method of Sedmak [75], with bovine serum albumin as the standard. SDS-PAGE was carried out to visualize and determine protein purity as previously described [76].

The molecular weight, theoretical pI, and amino acid frequencies were analyzed using ProtParam (http://web.expasy.org/protparam/ accessed on 11 November 2021).

### 4.5. Determination of Enzyme Activity and Kinetic Parameters of MrAII

L-asparaginase catalytic activity was measured with Jasco J-815 circular dichroism spectrometer (Jasco, Tokyo, Japan) with a temperature-controlled cell, as previously described [77]. Samples were prepared by mixing L-asparagine 1–40 mM and MrAII solution 0.03–0.035 mg/mL. The reactions were performed in quartz cuvettes (volume 300 μL, pathlength 1 mm). The time-dependent ellipticity was recorded at 210 nm. Specific enzyme activity was expressed in U/mg protein.

L-glutaminase and D-asparaginase activities were measured by the same procedure using L-glutamine or D-asparagine as substrates, respectively.

The kinetic parameters of MrAII were determined in borate buffer (0.025 M, pH 9.3) at 70 °C with L-asparagine as substrate. The observed data were fitted to the Michaelis–Menten equation, and the kinetic constants K_M_ and V_max_ were calculated from Lineweaver–Burk plots [34].

### 4.6. Effect of Temperature and pH

The activity at different temperatures and pH levels was studied for the purified enzyme.

For the optimum temperature, the enzyme activity profile was analyzed at different temperatures, ranging from 45 to 80 °C, with 5 °C increments. Additionally, MrAII activity was measured at 37 °C. The mixture was assayed in borate buffer (0.025 M, pH 9.3).

The thermal stability of L-ASNase from *M. roseus* was determined by detecting the residual activity of the enzyme that had been preincubated at different temperatures, ranging from 40 to 70 °C, with 10 °C increments, in borate buffer (0.025 M, pH 9.3).

The optimum pH was determined by assessing the enzyme activity of different pH levels at 70 °C in five buffer systems. Namely, sodium acetate (0.05 M, pH 4.0–6.0), sodium phosphate (0.05 M, pH 6.0–7.0), Tris–HCl buffer (0.05 M, pH 7.0–9.0), borate (0.025 M, pH 9.3), and glycine–NaOH buffer (0.05 M, pH 9.5).

### 4.7. Chemical Denaturation Studies and Effect of Various Metal Ions

Enzyme stability was investigated after 1 h incubation in borate buffer (0.025 M, pH 9.3) in the presence of 0–8.0 M urea. The activity of MrAII examined at 70 °C in the absence of urea was taken as 100%. The measured activities were compared with the activity of the enzyme without urea addition under the same conditions.

The effects of metal ions on MrAII activity were investigated in the presence of various cations (Ni^2+^, Cu^2+^, Mg^2+^, Zn^2+^, Ca^2+^, Fe^3+^) and EDTA. Salts NiCl_2_, CuSO_4_, MgCl_2_, ZnCl_2_, CaCl_2_, FeCl_3_ and EDTA were added at a concentration of 10 mM. The enzyme activity was assayed at 70 °C and pH 9.3 (in 0.025 M borate buffer) by adding L-asparagine and the corresponding metal ion(s) or EDTA. The entire procedure was triplicated.

The activity without any metal ion addition was set to 100%. The measured activities were compared with the activity of the enzyme without metal ion or EDTA addition under the same conditions.

### 4.8. Determination of Cytotoxic Activity

Human chronic myelogenous leukemia K562, acute T cell leukemia Jurkat, prostate carcinoma LnCap, and ovary adenocarcinoma SCOV-3 cell lines were grown in RPMI-1640 medium. WI-38 normal human fibroblasts were grown in DMEM medium and used as normal control adhesive cells.

The study involving normal human CD4+ T cells was approved by the Ethical Committee at the Institute of Biomedical Chemistry; written informed consent was obtained from all participants. Blood from healthy 18–25-year old donors (n = 4) was collected in Vacuette K3EDTA tubes (Greiner Bio-One, Kremsmünster, Austria). Fresh peripheral blood mononuclear cells (PBMCs) were isolated using Lympholite-H (Cedarlane, Burlington, Ontario, Canada) density gradient centrifugation. CD4+ T cells were purified from PBMCs using CD4+ Human Isolation Kit (Miltenyi Biotec, Bergisch Gladbach, Germany) according to the manufacturer’s instructions. CD4+ T cells were seeded at 5 × 10^5^ cell/mL and cultured in 25 cm^2^ flasks in RPMI 1640 cell medium supplemented with 10% FBS, 5 µg/mL anti-CD28, 5 µg/mL anti-CD3 mAbs, and 100 U/mL rHu IL-2 [78]. All cell lines were cultivated in 5% CO_2_/95% air in a humidified atmosphere at 37 °C, and tested for mycoplasma contamination before the experiment using the Mycoplasma Detection Kit PlasmoTest™ (InvivoGen, San Diego, CA, USA).

To test acute toxicity, cells were cultivated for 72 h in 96-well plates (TPP, Trasadingen, Switzerland), in the presence of the enzyme within the range of concentrations 1–100 U/mL, and cell viability was tested by measuring the conversion of the tetrazolium salt, 3-(4,5-dimethyl-thiazol-2-yl)-2,5-diphenyltetrazolium bromide to formazan (MTT test). IC_50_ and IC_90_ values (the concentration of the enzyme where the response was reduced by 50% and 90%, respectively) were calculated from the curve-fitting equations [79]. 

To measure apoptosis, incubated cells were dissociated using trypsin, resuspended in PBS and incubated with Annexin V-FITC and propidium iodide (PI) from a FITC Annexin V/Dead Cell Apoptosis kit (Life Technologies, Carlsbad, CA, USA) according to the manufacturer’s protocol. The counting of 5 × 10^4^ cells at each point was performed by flow cytometry with a MACS Quant Analyzer 10 (Miltenyi Biotec, Bergisch Gladbach, Germany), as we previously described [80].

### 4.9. Statistical Analysis

The experimental data are expressed as the mean value ± standard error, calculated from three parallel experiments. The statistical analysis was performed by one-way analysis of variance (ANOVA), using Microsoft Excel (version 2016).

For the measurement of cytotoxic activity, statistical analysis involving the Student’s t-test was implemented with the Statistica software (version 9.0, StatSoft, Tulsa, OK, USA). Differences described by *p* ≤ 0.05 were considered significant. The results are presented as the mean ± standard error of the mean (SEM).

## Figures and Tables

**Table 1:** Purification yield of recombinant type II L-asparaginase from *M. roseus* (MrAII).

Purification Step	Total Protein, mg	Total Activity, U	Specific Activity, U/mg	Yield, %	Purification Fold
Crude enzyme	205.0	109,300.0	533.2	100.0	-
Purified enzyme	88.0	82,572.0	1530.0	75.5	2.9

The purification factor was defined as the ratio between the specific activity of the purified enzyme and enzyme in the crude extract sample.

**Table 2:** IC_50_ and IC_90_ values of MrAII for cancer and normal cell lines.

Cell Line	IC_50_, U/mL	IC_90_, U/mL
K562	3.0	14.8
Jurkat	10.5	25.2
LnCap	15.8	46.4
Scov-3	12.1	39.4
Normal CD4+	44.5	96.3
WI-38	39.5	264.7

## Data Availability

The data presented in this study are contained within the article.

## Supplementary Materials

The following are available online at https://www.mdpi.com/article/10.3390/ijms222413632/s1.
