# Supplementary material for: Highly Active Thermophilic L-Asparaginase from Melioribacter roseus Represents a Novel Large Group of Type II Bacterial L-Asparaginases from Chlorobi-Ignavibacteriae-Bacteroidetes Clade
Source: Int J Mol Sci. 2021 Dec 20;22(24):13632. doi: 10.3390/ijms222413632 (PMC8709496; doi:10.3390/ijms222413632)

Supplementary Material

**Figure S1**. Sequence alignment of type III L-asparaginase from *M. roseus* (MrAIII) (WP_014855981.1), marked with yellow box, with characterized plant-type L-asparaginases. Conserved catalytic residues are shown in green

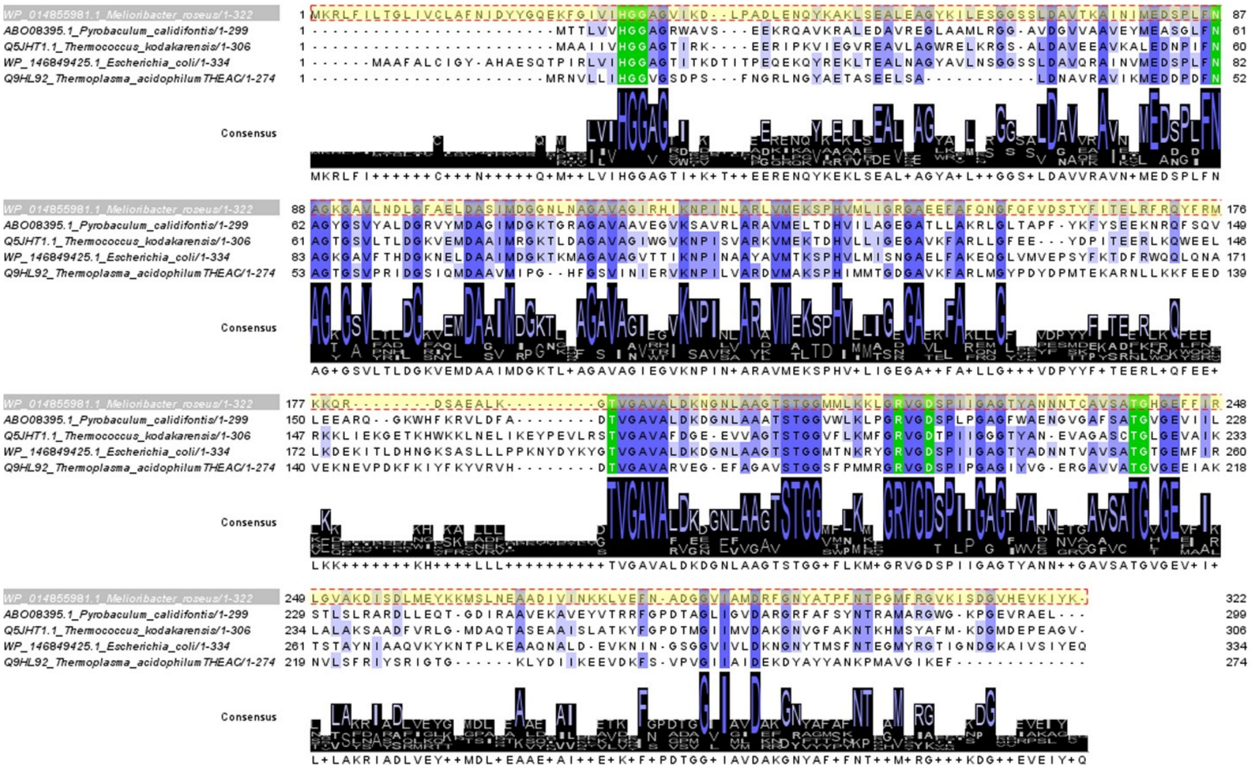

**Figure S2.** Cytotoxic activity of MrAII against cancer cells. Cancer and normal cells were cultivated for 72 h in the presence of different concentrations of the enzyme.

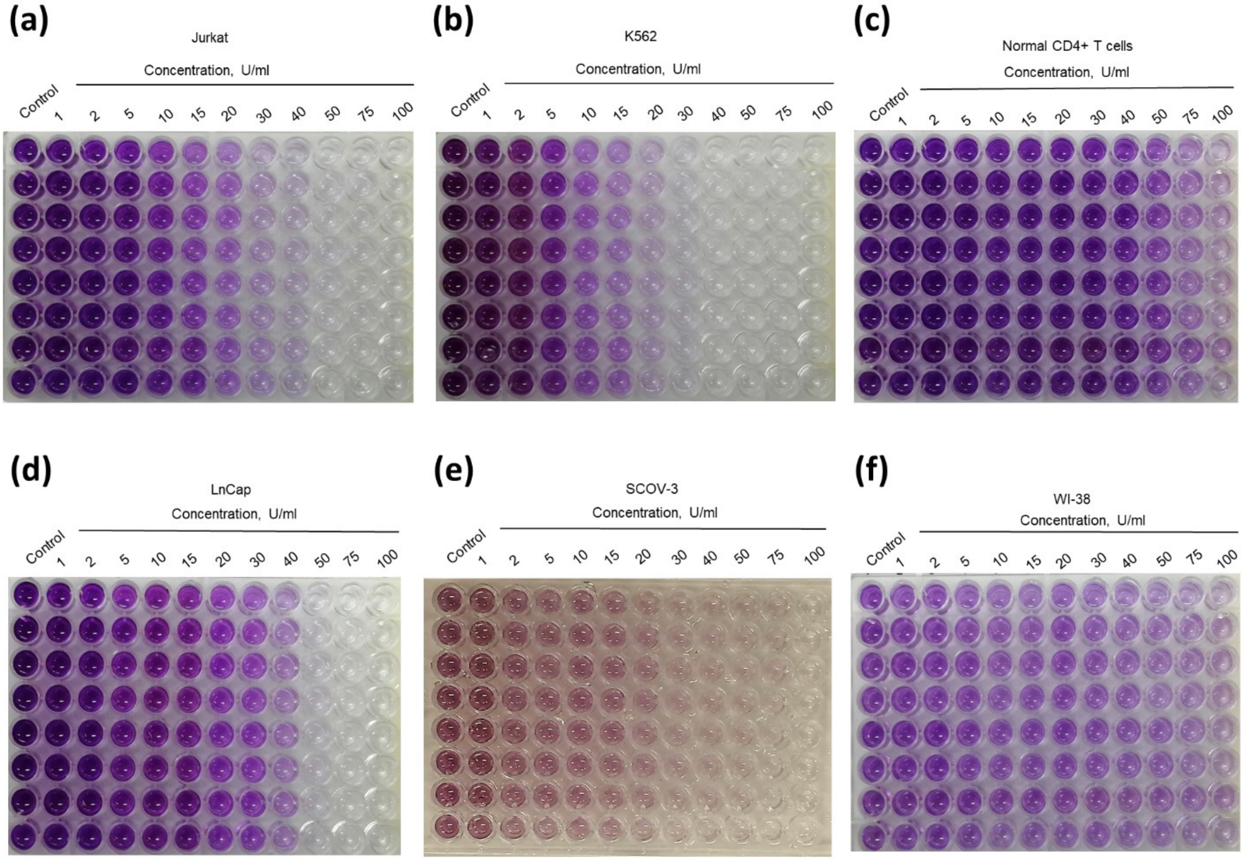

Supplement: Supplementary file 1 [file ijms-22-13632-s001.zip › ijms-1499929-supplementary.pdf]
